# Storage, fertilization and cost properties highlight the potential of dried microbial biomass as organic fertilizer

**DOI:** 10.1111/1751-7915.13554

**Published:** 2020-03-16

**Authors:** Janne Spanoghe, Oliver Grunert, Eva Wambacq, Myrsini Sakarika, Gustavo Papini, Abbas Alloul, Marc Spiller, Veerle Derycke, Lutgart Stragier, Harmien Verstraete, Koen Fauconnier, Willy Verstraete, Geert Haesaert, Siegfried E. Vlaeminck

**Affiliations:** ^1^ Research Group of Sustainable Energy, Air and Water Technology (DuEL) Department of Bioscience Engineering University of Antwerp Groenenborgerlaan 171 2020 Antwerpen Belgium; ^2^ Greenyard Horticulture Belgium NV Skaldenstraat 7a 9042 Gent Belgium; ^3^ Department of Plants and Crops Faculty of Bioscience Engineering Ghent University V. Vaerwyckweg 1 9000 Gent Belgium; ^4^ Avecom NV Industrieweg 122P 9032 Wondelgem Belgium; ^5^ AgrAqua Rooigemstraat 16 9860 Oosterzele Belgium; ^6^ Center for Microbial Ecology and Technology Faculty of Bioscience Engineering Ghent University Coupure Links 653 9000 Gent Belgium

## Abstract

The transition to sustainable agriculture and horticulture is a societal challenge of global importance. Fertilization with a minimum impact on the environment can facilitate this. Organic fertilizers can play an important role, given their typical release pattern and production through resource recovery. Microbial fertilizers (MFs) constitute an emerging class of organic fertilizers and consist of dried microbial biomass, for instance produced on effluents from the food and beverage industry. In this study, three groups of organisms were tested as MFs: a high‐rate consortium aerobic bacteria (CAB), the microalga *Arthrospira platensis* (‘Spirulina’) and a purple non‐sulfur bacterium (PNSB) *Rhodobacter* sp. During storage as dry products, the MFs showed light hygroscopic activity, but the mineral and organic fractions remained stable over a storage period of 91 days. For biological tests, a reference organic fertilizer (ROF) was used as positive control, and a commercial organic growing medium (GM) as substrate. The mineralization patterns without and with plants were similar for all MFs and ROF, with more than 70% of the organic nitrogen mineralized in 77 days. In a first fertilization trial with parsley, all MFs showed equal performance compared to ROF, and the plant fresh weight was even higher with CAB fertilization. CAB was subsequently used in a follow‐up trial with petunia and resulted in elevated plant height, comparable chlorophyll content and a higher amount of flowers compared to ROF. Finally, a cost estimation for packed GM with supplemented fertilizer indicated that CAB and a blend of CAB/PNSB (85%/15%) were most cost competitive, with an increase of 6% and 7% in cost compared to ROF. In conclusion, as bio‐based fertilizers, MFs have the potential to contribute to sustainable plant nutrition, performing as good as a commercially available organic fertilizer, and to a circular economy.

## Graphical abstract

Microbial fertilizer performed equal or even better in the fertilization of petunia and parsley. Microbial fertilizers showed promising quality which was preserved during storage. CAB and a blend of CAB/PNSB are the most cost‐effective fertilizer alternatives with an increase of 6 and 7% in cost price.

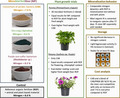

## Introduction

On a global scale, the agro‐industry is expanding due to the growth of the world’s population, which is expected to reach almost 10 billion by 2050 (Food and Agriculture Organization of the United Nations, 2017). To ensure food security and sustainable agriculture and horticulture, adequate plant nutrition and hence fertilization is indispensable. In agriculture, the fertilizers are applied directly onto the fields to produce arable crops (e.g. grains, potatoes). In horticulture, these fertilizers are applied to the soil or added to growing media (GM) for an effective production of fruits, vegetables and ornamental plants under controlled conditions (Gruda, 2009; Dixon and Aldous, 2014). In Flanders, the production value of the horticultural market is 2.2 times higher than the arable crop market in agriculture (Platteau and Van Bogaert, 2014).

The global demand for fertilizers amounts to an estimated 110 million tons (Mt) of N, 47.0 Mt P_2_O_5_ and 37.5 Mt K_2_O per year (Agriculture Production & International Trade and Market Intelligence Services, 2019; Food and Agriculture Organization of the United Nations, 2019). The vast majority of these fertilizers is supplied in the form of inorganic, ‘synthetic’ nutrients. Organic fertilizers, which are typically bio‐based, containing recovered resources from agro‐industry (byproducts, residues, and side and waste streams), only made up an estimated 5% of the total fertilizer market value in 2019 (Statistics Market Research Consulting Pvt Ltd, 2018; Brandessence Market Research and Consulting Pvt, 2019; Mordor Intelligence, 2020). However, the compound annual growth rate (CAGR) of the organic fertilizer market is estimated at 14.3%, which considerably exceeds the CAGR of 3.8% for the overall fertilizer market (Statistics Market Research Consulting Pvt Ltd, 2018; Mordor Intelligence, 2020).

Marketed organic fertilizers are typically solid and based on animal or plant materials (Sonneveld and Voogt, 2009), such as blood meal, cocoa shells, animal manures and soybean meal, amongst others (Wuang *et al.*, 2016). All organic fertilizers have in common that they release the nutrients gradually since the major part of the N, and P is bounded in complex molecules such as proteins and is released by decay or through decomposition by the microbial community associated with the soil or GM (Grunert *et al.*, 2016a, 2016b). Organic fertilizers may offer appealing benefits for plant and soil health, and the environmental footprint of plant production. This slow‐release characteristic results in a decrease of N leaching losses. Furthermore, the organic fertilizers can increase the organic matter content of the soil, which improves the exchange capacity of nutrients, promotes soil aggregates, increases soil water retention and buffers the soil against acidity, alkalinity, salinity, pesticides and toxic heavy metals (Chen, 2006; El‐Haggar, 2007; Paungfoo‐Lonhienne *et al.*, 2012).

Microbial fertilizers (MFs) constitute an emerging type of organic fertilizers composed of dried microbial biomass. As other organic fertilizers, MF is a direct source of plant macronutrients such as nitrogen, phosphorus, and potassium (N/P/K). The MF serves as a substrate to the microbiome present in the growing medium or soil, mineralizing the supplemented biomass, thereby rendering nutrients available for plant growth. This is not to be confused with a biofertilizer which contains active cells that can act as biocatalysts and contributes to the microbiome activity of the GM or the soil. A common application of these active cells is to render nutrients in the rhizosphere available to the plant roots, thereby providing an indirect fertilizer function. Other known application relate to bio‐stimulation, bio‐fortification and nutrient fixation (Sakarika *et al.*, 2020).

Microbial biomass, whether for use as MF or as microbial protein (single‐cell protein), can be efficiently produced in bioreactors, converting up to 100% of the inorganic nutrients to biomass, thus minimizing/avoiding environmental emissions (Verstraete *et al.*, 2016). The microbial biomass can also capture and concentrate dissolved nutrients from dilute side streams, and as such assist in local nutrient loop closure, through rendering an additional recovered source of nutrients available (Doucha *et al.*, 2005; Lee *et al.*, 2015; Alloul *et al.*, 2019).

Effluents from the food and beverage sector (i.e. potato processing, beer brewery) are not well explored as a source for bio‐based fertilizer production, even though they often contain substantial amounts of N, P and K (Falletti *et al.*, 2015). To comply with environmental regulations, nitrogen and phosphorus must be removed, avoiding eutrophication and oxygen depletion in the receiving water bodies. However, the removal process generally leads to the loss of these nutrients either to the air (i.e. N_2_ and N_2_O) or to waste sludge (i.e. P precipitated with iron or aluminium; N and P in microbial biomass). Nutrient recovery and reuse is mostly limited to applying some of the resulting biosolids on farmland, mostly as a slurry, with low nutrient release efficiencies (Kanagachandran and Jayaratne, 2006; DC water, 2020). There is tremendous improvement potential for the recovery and reuse of valuable nutrients from agro‐industrial side streams to provide the much needed fertilizers. While recovery as refined mineral compounds (e.g. struvite, ammonium sulfate) is technologically ready for implementation (Grunert *et al.*, 2019), the currently associated costs may slow down market entry (De Vrieze *et al.*, 2019).

**Fig. 1:**
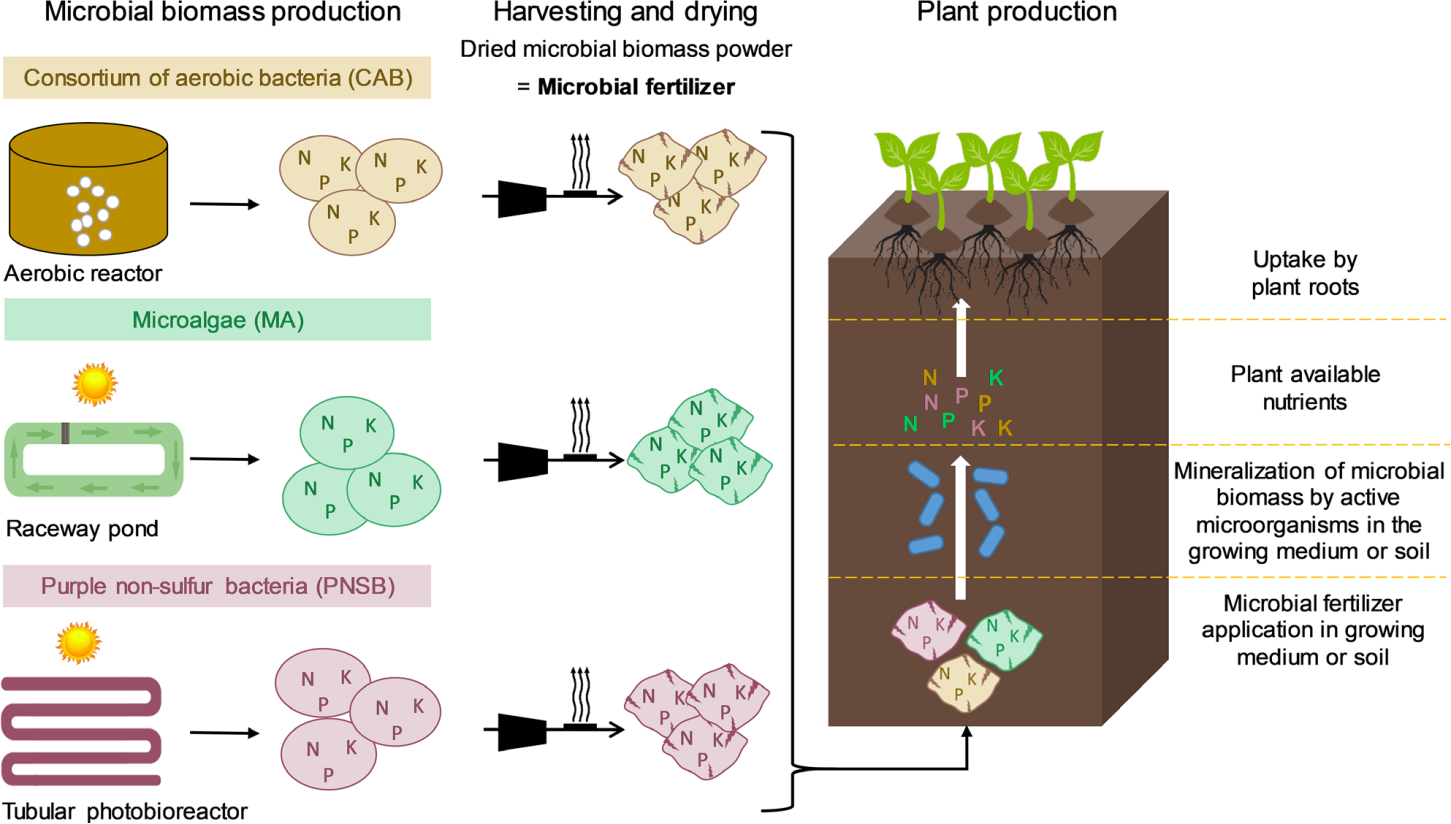
Conceptual scheme of the production of three metabolic types of microbial fertilizer (MF, dried microbial biomass powder), e.g. consortium of aerobic bacteria (CAB), microalgae (MA) and the purple non‐sulfur bacterium (PNSB). Subsequently, it is shown how MFs are applied in growing media or soils to produce plants.

In this study, three metabolic types of MF will be discussed: a consortium of aerobic bacteria (CAB), the microalga *Arthrospira platensis* (Spirulina) and the purple non‐sulfur bacterium (PNSB) *Rhodobacter* sp. Figure 1 presents the conceptual scheme of MF production and application. The microbial biomass is ideally produced in high‐rate systems (maximum production, high N content) at a low solid retention time (young biomass), to assimilate high nutrients levels, after which it is harvested and dried (down to around 10% moisture content). Spiller *et al. *(2019) provides a more detailed description of the processes and mass balances used to produce these three metabolic types of MF on potato wastewater. In terms of assessing fertilizer performance, N/P/K content, mineralization rate and fertilization effects are important parameters. As with other organic fertilizers, success relies on an active GM/soil microbiome (Grunert *et al.*, 2016a, 2016b).

Despite a strong research interest in producing nutrient‐rich biomass, the literature on applying dried microbial biomass as fertilizer is scarce and lacks a systematic approach. Typically, one metabolic type of MF is investigated at non‐comparable nutrient dosages, often with only an inorganic rather than an organic fertilizer as control, or without positive fertilization control. Furthermore, storage behaviour, mineralization patterns and economic aspects are not addressed. For CAB, no fertilization tests are available on biomass with a low age, i.e. expected to have high nutrient content and slow release pattern. Kanagachandran and Jayaratne (2006) performed a study on sundried CAB grown at relatively high cell age on brewery wastewater, combined with compost. Germination and plant growth of pumpkin seeds and chili seeds was stimulated when these CAB were added to the compost. For Spirulina, promising results were demonstrated in leafy vegetables, compared to an inorganic fertilizer as control (Wuang *et al.*, 2016). This Spirulina biomass was grown on aquaculture wastewater, but no additional information was available on drying procedure. For PNSB, freeze‐dried *Rhodopseudomonas* spp. or *Rhodobacter* spp. gave positive results for the production of tomatoes (Kondo *et al.*, 2010), pakchoi (Wong *et al.*, 2014), spinach (kondo *et al.*, 2008) and rice (Kobayashi and Haque, 1971). These PNSB studies either used an inorganic fertilizer or no fertilizer as a control.

The novelty in this research is the systematic comparison of novel types of MF (CAB, Spirulina and *Rhodobacter* sp.), in which a reference organic fertilizer (ROF) was used as a practical benchmark (positive control). Focus was on the key aspects of investigating the feasibility of this new value chain, ranging from nutrient levels, over shelf‐life of dried fertilizer and industrial storage after blending with growing medium, to fertilization properties on two crops and a cost estimation. Furthermore, to the authors’ knowledge, a number of these aspects have not been reported before for microbial fertilizers, including its use to produce herbs and ornamental plants, the compositional dynamics of stored products and an economic analysis.

## Results

### Chemical characterization of the MF and ROF

In Table 1, the nitrogen, phosphorus and potassium content are shown for each MF and ROF based on their weight as dried product. The nitrogen content for all MF and ROF is comparable, while for phosphorus and potassium, there is a larger variability between the fertilizers. For all following experiments, the applied dose of MF or MF blend was normalized according to the respective nitrogen concentration.

**Table 1:** Nitrogen (N), phosphorus (P) and potassium (K) content of the reference organic fertilizer (ROF), consortium of aerobic bacteria (CAB), Spirulina (microalga) and *Rhodobacter* sp. (PNSB) used in this study.

Category	Tag	g/100 g dried product		
		Nitrogen (N)	Phosphorus (P)	Potassium (K)
Plant‐ and animal‐based material	ROF	8.0	2.2	5.0
Aerobic heterotrophic bacteria	CAB	7.0	0.7	2.0
Photoautotrophic (Microalga)	Spirulina	8.6	0.3	0.7
Photoheterotrophic (PNSB)	*Rhodobacter* sp.	8.5	2.4	0.5

### Performance during fertilization trials

A first trial was set up with parsley (*Petroselinum crispum* cv. Grüne Perle) including all MFs as well as a blend of the three MFs to determine the performance. The MFs and ROF were always mixed in a growing medium (GM) in which phosphorus and potassium were present in sufficient amounts (minimum of 26 mg P and 190 mg K l^−1^ GM) to avoid deficiency symptoms and growth limitations (Straver *et al.*, 1999).

#### Parsley

**Fig. 2:**
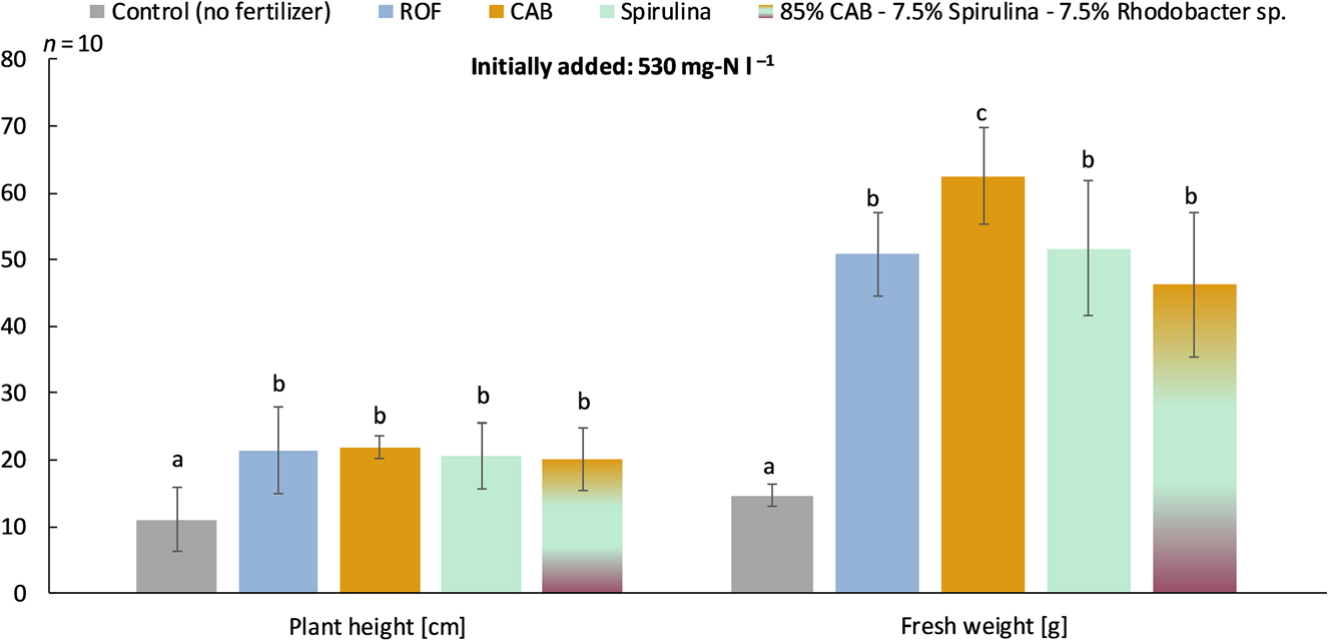
Plant height and fresh weight of parsley after 49 days in relation to the different fertilizers (530 mg N l^−1^ GM). The error bars indicate the standard deviation of the different treatments (*n* = 10). Abbreviations: GM, growing medium; ROF, reference organic fertilizer; CAB, consortium of aerobic bacteria; Spirulina, Microalga; *Rhodobacter* sp., purple non‐sulfur bacterium control, no fertilizer addition; significant differences between treatments are indicated by letter codes per harvesting moment.

The GM was supplemented with the MFs and ROF to obtain a final concentration of 530 mg N l^−1^ GM. CAB, Spirulina and ROF were tested individually, and there was also a fertilizer blend of 85% CAB, 7.5% Spirulina and 7.5% *Rhodobacter* species. Figure 2 illustrates the results of the parsley growth test. The plant height was equal for all MFs (and the blend) compared to ROF, and they were all significantly outperforming the control with no added fertilizer. For fresh weight, the use of CAB resulted in the highest value, differing significantly from the other MFs and ROF. Due to the promising results of CAB in this trial, CAB was used in a follow‐up trial with petunia (*Surfinia* cv. Purple*).*

#### Petunia

**Fig. 3:**
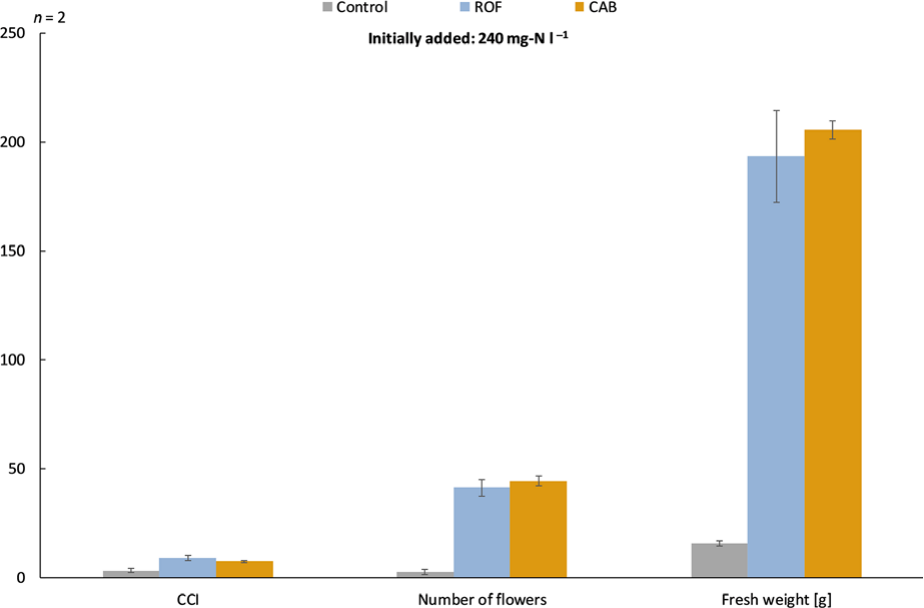
Growth performance of petunia measured in chlorophyll content index (CCI), number of flowers and fresh weight for reference organic fertilizer (ROF) and consortium of aerobic bacteria (CAB) that was added to the growing medium in a concentration of 240 mg‐N l^−1^. In the control, no fertilizer was added.

A negative control (no extra nitrogen supplied) was compared to supplementation of 240 mg N l^−1^ GM of ROF and CAB*.* Figure 3 shows the plant performance of petunia grown on ROF and CAB. Both fertilizers provoked good flowering with 31‐50 flowers per plant as compared to a negative control (no fertilizer added). According to the chlorophyll content index (CCI) measurements, a medium plant colour was found for ROF (CCI = 9.10) and CAB (CCI = 7.35), while the control showed yellow leaves (CCI = 3.20). In addition, the fresh weight of the above ground petunia plant was measured, resulting in the highest fresh weight (206 g) for CAB, while ROF resulted in a good fresh weight (193 g) and the control had the lowest fresh weight (16 g).

General plant health was visually assessed after 26 days and 89 days and given a score from 1–10 (based on Fig. S2). According to the results of the trial, the general health of plants fertilized with respectively ROF, CAB and control was categorized as good (8–10), medium (6–7) and bad (≤ 5) respectively. Root development (based on Fig. S2) was assessed at 71 and 92 days after the start of the trial. The plants fertilized with CAB obtained a good rooting score (> 4), while ROF and the control treatment had a poor score for rooting (≤ 2.5).

### Mineralization

#### Nitrogen dynamics in packed growing medium

**Fig. 4:**
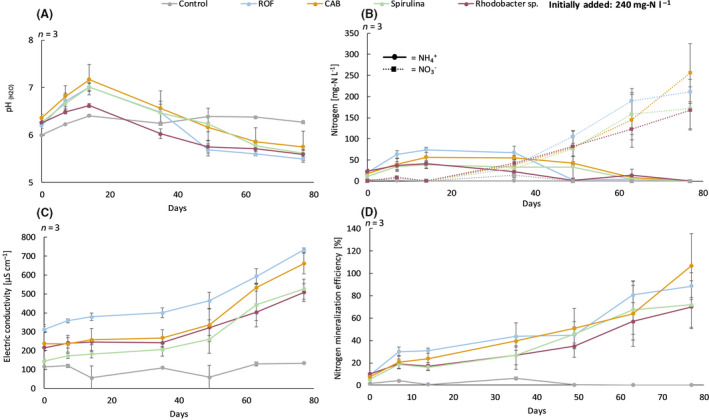
Nitrogen dynamics, pH and electric conductivity (EC) evolution in the growing medium during 77 days of incubation: (A) evolution of the pH over time, (B) evolution of the ammonium nitrogen and nitrate nitrogen content per treatment, (C) evolution of the electrical conductivity over time and (D) percentage of mineralized nitrogen in function of time, with 240 mg N l^−1^ as 100%. Mean values per object are presented with error bars representing their resp. standard deviation. Abbreviations: ROF, reference organic fertilizer; CAB, consortium of aerobic bacteria; Spirulina, *Arthrospira platensis*; *Rhodobacter* sp., purple non‐sulfur bacterium; control, no fertilizer addition. Different letters next to the dots indicate significant differences at *P* = 0.05.

The GM was supplemented with 240 mg N l^−1^ GM using the three MFs and ROF, next to a control without fertilizer. Per treatment, three 40‐l bags (EN12580) were filled and stored under industrial production conditions from July 2017 until the end of October 2017. The GM was stored on pallets to avoid contact with soil and moisture and was covered with opaque plastic to minimize potential sun damage and moisture intrusion. The ammonium and nitrate content, mineralized nitrogen, pH and the electrical conductivity (EC) during 77 days of incubation are visualized in Figure 4A–D. The mineralization of organically derived nitrogen had a considerable impact on the pH of the GM (Fig. 4A). Within 14 days, there was a net pH increase of 0.2–0.4 pH units due to the ammonification of organic nitrogen compared to the control. After 14 days, the pH decreased due the appearance of H^+^ ions, attributable to subsequent nitritation and nitratation. CAB, Spirulina and ROF showed the largest net increases of the pH, while *Rhodobacter* sp. showed a comparable increase of the pH compared to the no‐fertilizer control. After 77 days, the pH dropped to 5.6, while the pH of the control GM levelled between 6.1 and 6.3. However, differences in pH of the GM containing the different microbial fertilizers were rather small in absolute terms.

From Figure 4B, it can be concluded that ROF had the highest increase in NH_4_^+^ –N content (≈ 80 mg l^−1^ or 33% of the supplied nitrogen) within 14 days compared to the other fertilizers (≈ 40 mg l^−1^ or 18% of the supplied nitrogen). Between 14 and 36 days, the nitrate concentration steadily increased and the highest NO_3_^−^–N concentrations were found for ROF, while *Rhodobacter* sp. remained the lowest. The nitrification rate can be calculated on the slope from 35–77 days and normalized for GM. The results for the nitrification rate in packed GM without plants are shown in Table 2.

**Table 2:** Nitrification rate of the reference organic fertilizer (ROF), consortium of aerobic bacteria (CAB), Spirulina (Microalga) and *Rhodobacter* sp. (PNSB) in the growing medium (GM) without and with plants (parsley).

	Nitrification rate in GM (mg N l^−1^ GM day^−1^)	
	Without plants	With plants (parsley)
ROF	4.36 ± 0.65 (*R* ^2^ = 0.96, *n* = 4)	28.41 (29.38; 427.14)
CAB	5.12 ± 0.81 (*R* ^2^ = 0.95, *n* = 4)	28.17 (12.43; 406.8)
Spirulina	3.61 ± 0.62 (*R* ^2^ = 0.94, *n* = 4)	31.08 (23.73; 458.78)
*Rhodobacter* sp.	2.99 ± 0.06 (*R* ^2^ = 1.00, *n* = 4)	N/A
85% CAB 15% Spirulina 15% *Rhodobacter* sp.	N/A	34.38 (20.34; 501.72)

Standard errors on the slope are given (for the parsley growth test *n* = 2, therefore no standard error is given; instead, the nitrate concentrations are given for day 14 and day 28 on which the rate is calculated). N/A = not available.

After 77 days, respectively, 89 ± 12%, 107 ± 29%, 72 ± 21% and 70 ± 18% of the applied nitrogen coming from ROF, CAB, Spirulina and *Rhodobacter* sp. was transformed into ammonium and nitrate (Fig. 4D). These variations in N‐release patterns are likely related to differences in chemical characteristics of the fertilizer blends, such as the C:N ratio and the N content. However, in absolute value and from a practical point of view, differences between the fertilizer treatments were relatively small. Evidently, the control contained only low amounts of ammonium nitrogen as well as nitrate nitrogen compared to all other treatments. In addition, Figure 4C shows the effect of the mineralization of the organic derived nitrogen on the electric conductivity (EC) over time. ROF has the highest EC from the start and the differences of 200 µS cm^‐1^ at sampling after 7, 14, 35, 49, 63 and 77 days were similar to those obtained at the start, compared to the MF. Also, the three MFs showed a comparable increase of EC over time.

#### Nitrogen dynamics during plant growth test

**Fig. 5:**
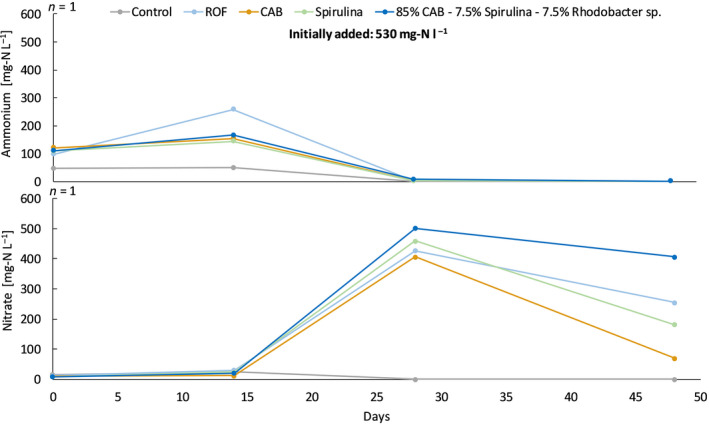
Nitrogen mineralization (ammonium and nitrate nitrogen) was measured over time in the growing medium respectively without fertilizer and with the different fertilizer combinations (ROF, reference organic fertilizer; CAB, consortium of aerobic bacteria; Spirulina, *Arthrospira platensis*; *Rhodobacter* sp., purple non‐sulfur bacterium; control, no fertilizer addition) at dose of 530 mg N l^−1^ GM.

During the parsley growth test, the evolution of ammonium and nitrate concentration in the GM was monitored as seen in Figure 5. In general, there was a fast increase in ammonium concentration during the first 14 days, which was totally eliminated within the next 14 days. Furthermore, ROF showed the fastest increase in ammonium concentration compared to the MFs. The nitrate concentration also showed similar trends for the different fertilizers. Nitrate concentrations started to increase after 14 days while ammonium was decreasing. The latter was confirmed by the pH and EC changes of the GM (Fig. S2) over time. A minimal nitrification rate was determined, as shown in Table 2 (based on two data points only).

### Shelf‐life of the microbial fertilizer

**Fig. 6:**
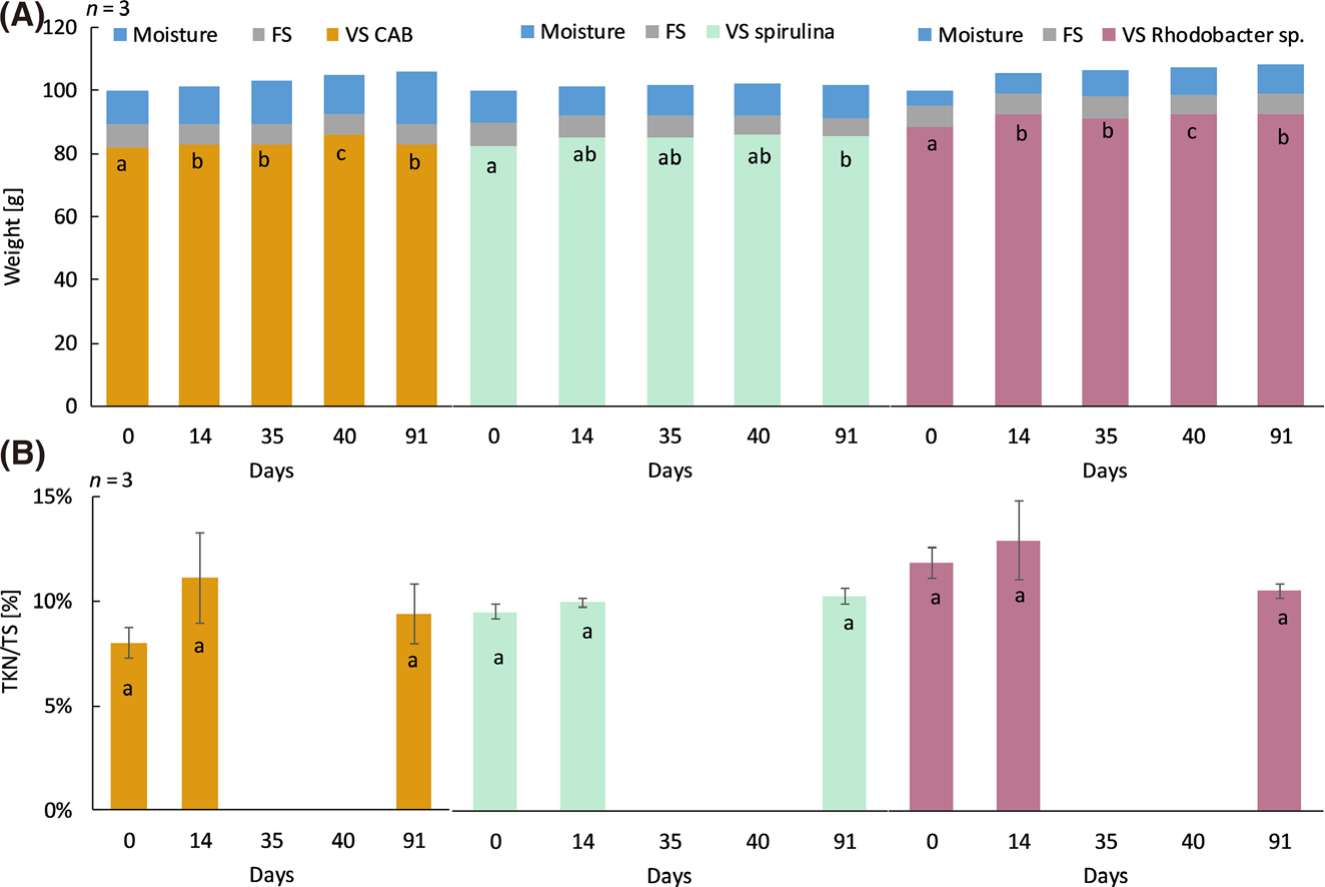
Evolution of the product composition throughout the storage test of the individual microbial fertilizers CAB (consortium of aerobic bacteria), Spirulina (a microalga); *Rhodobacter* sp. (a purple non‐sulfur bacterium). A. Absolute values of the volatile solids (VS), fixed solids (FS) and moisture content. The product is composed of moisture and total solids (TS), TS is in turn composed of organic (VS) and inorganic constituents (FS). Mean values per MF are presented with day 0 taken as 100 g of TS. The error bars (standard deviations) are not visible on the graph. The letters denote statistical differences between VS content of the biomass between the timepoints. B. Relative total Kjeldahl nitrogen (TKN) content, expressed per total solids. Mean values per MF are presented with the error bars (standard deviations). The letters denote statistical differences between TKN/TS content of the biomass between timepoints.

A storage test of 91 days (13 weeks) was conducted with each MF to determine the hygroscopic activity and the shelf‐life of the products, defined as the time required for 10% of the material to disappear (Drug product, FDA, 1987). In Figure 6A, the volatile solids (VS), fixed solids (FS) and moisture, making up the total solids (TS), are shown in absolute values for the three MF over the course of the storage test. Significant differences were found in the VS content over the period of 91 days, but they are not decreasing or increasing in a specific pattern. An increase in moisture content (hygroscopic activity) was seen for CAB and *Rhodobacter* sp., but stayed limited to a percentage increase of, respectively, 5% and 3%; for Spirulina, this was only 1%.

In Figure 6B, the total Kjeldahl nitrogen (TKN) content of the dry biomass (TS) is presented. The nitrogen content of the microbial fertilizer was monitored, since this is the most important factor that was tested in the elaborated storage test and the fertilization experiments. Over the course of the storage test (days 0–91), there was no significant difference in TKN/TS content.

### Cost assessments

**Fig. 7:**
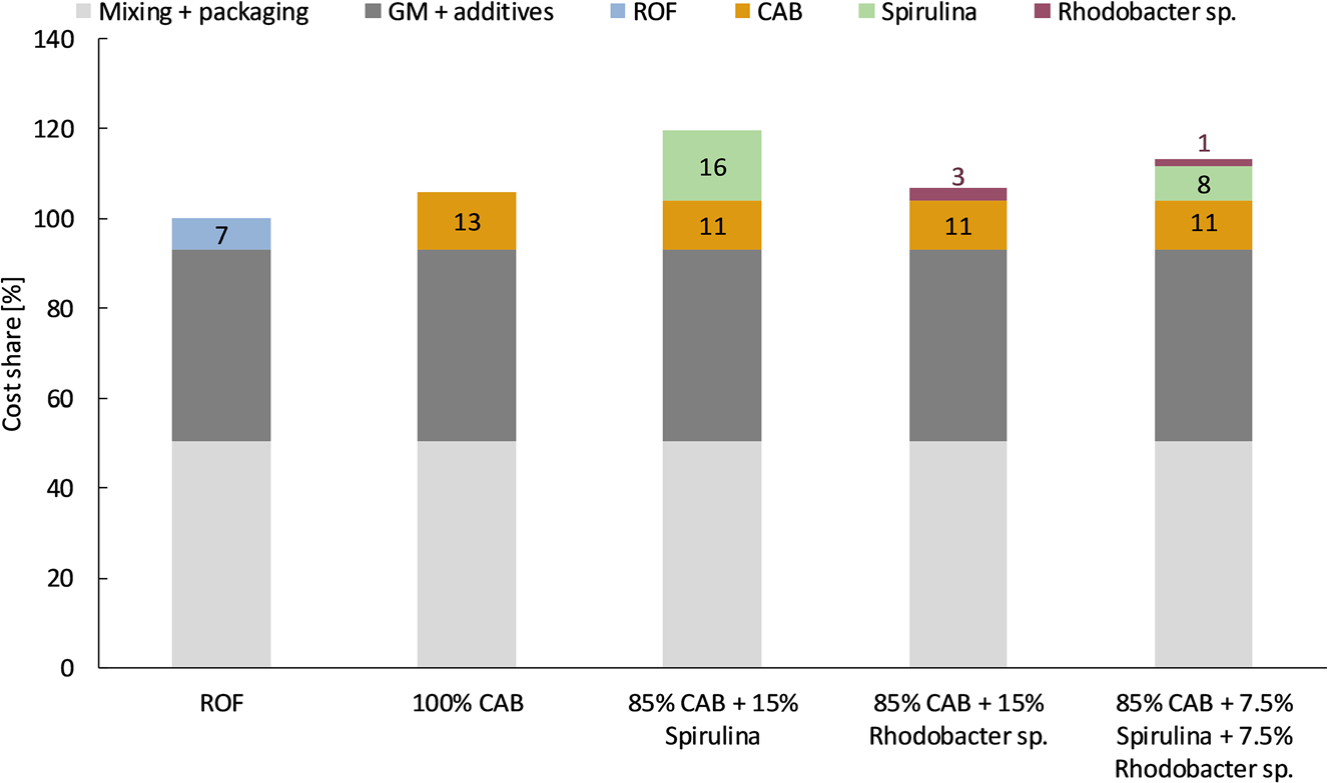
Cost assessments of packed growing medium supplemented with fertilizers (320 mg‐N l^−1^). The total cost for a packed bag supplemented with reference organic fertilizer (ROF) is taken as 100%. Abbreviations: GM, growing medium; ROF, reference organic fertilizer; CAB, consortium of aerobic bacteria; Spirulina, microalga; *Rhodobacter* sp., purple non‐sulfur bacterium.

A cost assessment was made for packed GM in bags of 40 litre, which were supplemented with fertilizers to reach a nitrogen content of 320 mg N l^−1^ GM. The total cost is composed of the GM with additives, the fertilizers and mixing/packaging cost. Figure 7 shows the results of the cost assessments for the different fertilizers and blends. The cost for packed GM supplemented with 100 % CAB appears to be only 6% more expensive than adding ROF (a standard commercial organic fertilizer). For the blends using the phototrophic organisms (Spirulina and *Rhodobacter* sp.), the increase in cost is 7–20%.

## Discussion

### Microbial fertilizer production and quality

Overall, the MFs showed comparable N and P quality with ROF (Table 1). The higher phosphorus content of *Rhodobacter* sp. can be explained by the phosphate accumulating capacities of PNSB (Hiraishi *et al.*, 1991; Lai *et al.*, 2017). In terms of drying conditions, as for other organic products subjected to heat processing (e.g. corn; Odjo *et al.*, 2015), a considerable degree of expertise may still need to be developed on this topic for cost‐effective microbial biomass drying in terms of heating rate and level, and their impact on the final quality of the product. Furthermore, the microbial community of CAB was not analyzed in this study, with the application in mind of using the powder product as a direct source of plant macronutrients (N, P, K). It is known that such communities are inherently dynamic and furthermore impacted by process conditions (Meerburg *et al.*, 2016; Langer *et al.*, 2019). Future research on fertilizers based on complex communities could benefit from understanding whether and which compositional changes may alter their fertilization properties. Alternatively, microbial biomass that is not dried and mostly viable, a so‐called biofertilizer, is known to perform indirect fertilization (e.g. biological N_2_ fixation), or possess bioactive compounds to promote plant growth and health (Shen *et al.*, 2019; Sakarika *et al.*, 2020). It is currently unknown whether bioactive compounds remain present and active after drying, and this research line should be further explored to include all growth‐promoting effects.

### Fertilization performance

Parsley growth showed an equal performance for all MFs (and their blends) when compared to ROF for plant height and fresh weight. These results suggest that the developed MFs have a nutrient release pattern close to the actual needs of the plant. However, CAB performed significantly better when the fresh weight of the parsley plants was applied as an evaluation parameter. The follow‐up experiment with the ornamental plant petunia (only performed for CAB) confirmed the better performance of CAB when compared to the ROF. CAB obtained the highest fresh weight (206 g) and a superior rooting score. The results for CCI and number of flowers showed similar trends for CAB and ROF.

### Mineralization behaviour

The MFs showed similar nitrogen mineralization patterns in the GM compared to ROF, i.e. mineralization rate as well as the ammonium and nitrate content. However, an advantage of the use of the MFs compared to ROF is the slightly lower ammonium concentrations during mineralization (without and with plants). Too high ammonium concentrations might cause ammonium toxicity, resulting in stunted growth, necrotic leaves, inhibition of the primary root growth, and, in severe cases, even plant death (da Silva *et al.*, 2016). Additionally, it was shown that there is a high correlation between the EC and nitrogen mineralization efficiencies (Fig. 4D), which is the sum of the ammonium and nitrate concentrations. The correlation coefficients between EC and nitrogen mineralization efficiency for ROF, CAB, Spirulina and *Rhodobacter* sp. were 0.96, 0.95, 0.96 and 0.98 respectively. Therefore, the EC could be used as a proxy for nitrogen mineralization.

All fertilizers showed lower nitrification rates during mineralization in the packed GM without plants compared to during the parsley growth (Table 2). The major difference in nitrification rate between the mineralization without and with plant growth (5.5–8.6 higher) can be explained by the better accessibility for oxygen and water in the pots used for the plant trial compared to the 40‐l storage bags. The mineralization of the MFs in the packed GM (industrial storage) showed that more than 70% of the organic N is converted to inorganic species after 77 days. It is therefore recommended to store GM and MF separate and only mix these when needed to align the slow‐release characteristic with the plant’s needs. Therefore, a storage test of the individual MFs was performed to determine its shelf‐life.

### Storage

The storage test showed slight hygroscopic activity (increase of 3–5% in moisture) for CAB and *Rhodobacter* sp. It is known that particle size distribution influences product properties, such as caking (i.e. clumping of biomass through the uptake of water). In a study on lactose powders (also an organic material), smaller particles showed higher moisture sorption and a greater caking tendency (Carpin *et al.*, 2017). The higher hygroscopicity of the CAB biomass might therefore be attributed to the smaller particle size of this end‐product (about 0.5 mm granular size). Spirulina and *Rhodobacter* sp. both had larger particle sizes. Some caking was noticeable in the sample bags of CAB over the course of the storage test. Additionally, the moisture content increase might be caused by the extra dry texture at the onset. *Rhodobacter* sp. had an initial dry weight of 95%, which is higher than necessary for the end‐product. To avoid moisture increases in the biomass, vacuum packaging for storing can be considered. Overall, the VS content and the TKN/TS content had not decreased by 10%, and thus, the shelf‐life of the biomass is at least 91 days.

### Cost estimations of microbial fertilizers

A cost assessment of the MF (and their blends) was made in comparison with the commercially available ROF. CAB proved to be a cost‐effective alternative for ROF, with only an increase in cost of 6%. The blends with phototrophic bacteria (i.e. Spirulina and *Rhodobacter* sp.) resulted in increases between 7 and 20%. However, phototrophic microorganisms (i.e. Spirulina and *Rhodobacter* sp.) are known to contain plant growth promoting substances such as phytohormones, vitamins, carotenoids and antifungal substances (Serdyuk *et al.*, 1993; Spolaore *et al.*, 2006). The activity and content of bioactive compounds that remains active after drying is unknown, but could have an added‐value in the MF. Future research should focus on the effect of these bioactive compounds in the MF, since this could justify the higher cost of the blends.

## Conclusions

The individual and blended microbial fertilizers were shown to have an equal fertilization, mineralization and storage performance as a state‐of‐the‐art organic fertilizer. This means that these microbial fertilizers can be a viable option to set up production chains for local production of organic fertilizers. Particularly, CAB and a blend of CAB/PNSB (85%/15%) appear to be cost‐effective alternatives, with only an increase of 6% and 7%, respectively, in cost.

## Experimental procedures

### Characterization of the microbial products and the reference organic fertilizer

All MFs were used as a dried powder (± 90% dry weight) and were characterized for their N/P/K content. This content is expressed as a weight percentage per amount of dried product. Total nitrogen content of all individual fertilizers was determined according to Dumas’ method (EN 13654‐2) with a Vario Max device (Elementar). K and P were extracted (1:5 v/v) in 0.5 M ammonium acetate buffered at pH 4.65 (with 96% acetic acid) and then determined by inductively coupled plasma (according to EC 2003/2003 BNL‐P‐2 and BNL‐K‐1, with an Interpid 2 XSP (Thermo)). Detailed information on the production of the microbial fertilizers can be found in Supplementary information. For all following experiments, the applied dose of MF or MF blend was normalized according to the respective nitrogen concentration.

### Fertilization performance

The growing medium (309 kg m^−3^) used in the fertilization experiments was composed of 20 vol.% black peat, 50 vol.% white peat, 20 vol.% coconut coir pith and 10 vol.% green waste compost. Lime (CaMg(CO_3_)_2_) with an acid‐binding capacity of 55 was added (3 kg m^−3^) to adjust the pH to acceptable plant levels, i.e. pH_(H2O) _= 5.5–6.0. Phosphorus and potassium were present in the GM in sufficient amounts (minimum of 26 mg P and 190 mg K l^−1^ GM) to avoid deficiency symptoms and growth limitations (Straver *et al.*, 1999).

#### Parsley

The performance of the three MFs was tested in a plant growth trial with parsley, *Petroselinum crispum* cv. Grüne Perle. The test was executed in 12‐cm‐diameter pots in a greenhouse over 49 days (14/2/2017–5/4/2017). The GM was supplemented with the MFs and ROF depending on their nitrogen concentration, to obtain a final concentration of 530 mg N l^−1^ of GM. CAB, Spirulina and ROF were tested individually and there was also a fertilizer blend of 85% CAB, 7.5% Spirulina and 7.5% *Rhodobacter* sp.

The experiment was elaborated as a randomized block design, with four blocks, five different fertilizer combinations and fifteen replications per fertilizer treatment of which 10 were measured. Each container, which was filled with 650 ml of GM, contained 60 seeds. Plants were top‐irrigated with water according to the need, hence avoiding nutrient leaching until the seeds fully emerged. Afterwards the plants were sub‐irrigated until the end of the experiment with tap water (EC = 0.4 µS cm^−1^). Until seed emergence, the greenhouse was ventilated when temperatures were higher than 22°C (day/night) and was additionally heated when temperatures were below 20°C. After seed emergence, the greenhouse was ventilated when temperatures were higher than 20°C (day/night) and additionally heated when temperatures were lower than 18°C up to the end of the experiment. The parasitic wasp *Encarsia formosa* was used to control glasshouse pests, such as *Trialeurodes vaporariorum*. As a performance indicator, plant height and fresh weight were measured on ten randomly chosen plants out of the fifteen replicates.

#### Petunia

The effect of one microbial fertilizer, i.e. CAB, on petunia (*Surfinia* cv. Purple*)* was assessed in a pot trial. A negative control (no extra nitrogen supplied) was compared to supplementation with 240 mg N l^−1^ GM of ROF and CAB*.* Three rooted cuttings of petunia (Raesplant, Destelbergen, Belgium) were planted with two replicates per treatment in 10 litre (EN12580) pots. The pots were placed randomly throughout a glasshouse (PCS, Ornamental Plant Research, Destelbergen, Belgium). Plants were sub‐irrigated with non‐acidified rain water (EC = 0.1 µS cm^−1^). During the growing period of 92 days (29/3/2017–29/6/2017), plant performance was followed. Flowers were counted at the end of the trial (29/6/2017). Leaf colour of petunia was assessed at the end of the trial using a chlorophyll (SPAD) meter (Opti‐Sciences, Opti‐200 Plus, Hudson, USA). The chlorophyll meter measures the optical absorbance in two different wavebands: 653 nm (chlorophyll) and 931 nm (near infra‐red) and has a measurement area of 9.52 mm (3/8″) diameter circle (71 mm^2^). The meter consists of a silicon photodiode with integral amplifier for absorbance measurement, power monitoring and temperature compensation. Chlorophyll measurements were carried out on the second fully developed leaf of each plant with two replicates. A high chlorophyll content index (CCI) indicates a greener leaf, which indicates that the plant is taking up the nutrients needed to grow well and being healthy. A yellow light‐green colour can be a sign of an imbalanced nutrient uptake and indicates that the plant is in stress. General plant health and root development were visually assessed on 24/4/2017 (after 26 days) and 26/6/2017 (after 89 days) and given a score from 1‐10 (Fig. S1). Greenhouse climatic conditions (temperature, humidity) were registered during the entire trial and were, respectively, 18.5°C and 64.9 %on average.

### Mineralization: nitrogen dynamics in packed growing medium and during plant growth

The GM was supplemented with 240 mg N l^−1^ GM using the three MFs and ROF, next to a control without fertilizer. Per treatment, three bags (EN12580) were filled with 40 litre of GM and stored outside under industrial production conditions from July 2017 until the end of October 2017. The GM was stored on pallets to avoid contact with soil and soil moisture and was covered with opaque plastic to minimize potential sun damage and moisture intrusion. The average relative humidity and temperature over the period were, respectively, 75 (± 5)% and 16.0 (± 2.7)°C (Koninklijk Meteorologisch Instituut België, 2019). After 0, 7, 14, 35, 49, 63 and 77 days, samples of the GM were taken and three replicates per treatment were analyzed for ammonia nitrogen and nitrate nitrogen (according to method CMA/2/IV/C.7) to determine the nitrogen mineralization. Additionally, the pH and the electrical conductivity (EC) of the GM were determined in a 1:5 v/v water extract according to EN 13038 and EN 13037 respectively. Additionally, the nitrogen mineralization profile was determined during the parsley growth test. The nitrate and ammonium concentration were determined every two weeks during the whole cultivation period (14/2/2017–3/4/2017) according to method CMA/2/IV/C.7.

### Storage experiment

A storage test of 91 days (13 weeks) was conducted with each individual MF to determine the hygroscopic activity and the shelf‐life of the products. The shelf‐life is defined as the time required for 10% of the material to disappear and thus expire (Drug product, FDA, 1987). Fertilizers were stored in multiple sample bags (LDPE, 70 × 100 mm) in a dark room controlled at a temperature of 20°C and 60% relative humidity. At day 0, 14, 35, 56 and 91, a sample bag of each microbial fertilizer was sacrificed for determination of total solids (TS), volatile solids (VS), moisture and total Kjeldahl nitrogen (TKN). TS, VS and moisture were determined by weight difference of a sample before drying, after drying at 105°C (TS) and incinerating at 550°C (VS). The fixed solids (FS), inorganic (mineral) matter, are the difference between TS and VS. These analyses were performed according to Standard Methods 2540G (Eaton *et al.*, 2005). TKN was analysed according to standard methods (4500‐Norg B; Eaton *et al.*, 2005).

### Cost assessment

A cost assessment was made for packed GM in bags of 40 l, which were supplemented with fertilizers to reach a nitrogen content of 320 mg N l^−1^ GM. The total cost is composed of the GM with additives, the fertilizers and mixing/packaging cost. The exact composition of the GM (309 kg m^−3^) is 20 vol.% black peat, 50 vol.% white peat, 20 vol.% coco coir pith and 10 vol.% green garden compost RHP (‘Regeling Handels Potgronden’ – regulation for trading potting soils). Lime (CaMg(CO_3_)_2_) with an acid‐binding capacity of 55 was added (3 kg m^−3^) to adjust the pH to acceptable plant levels, i.e. pH_H2O_ = 5.5–6.0. Additional trace elements (B, Cu, Fe, Mn, Mo, Zn) were added at 150 g m^−3^ (Micromax Premium, ICL, The Netherlands). The cost calculation for GM, additives, ROF and mixing/packaging was provided by the company Greenyard (Sint‐Katelijne‐Waver, Belgium). These were 15.07 € m^−3^ GM, 0.24 € kg^−1^ additives, 0.61 € kg^−1^ ROF and some fixed and variable costs for mixing, packaging, a production cost depending on a bagging key and a fixed production costs per bag, the production costs and a fixed overhead cost, respectively (more detailed information can be found in the Table S1). The costs for CAB were communicated by the company Avecom (Wondelgem, Belgium) and the Spirulina cost by the company AgrAqua (Scheldewindeke, Belgium) and were, respectively, 1 and 10 € kg^−1^ dried product. The production cost of *Rhodobacter* sp. was based on an economic estimation based on a raceway pond, yielding a cost a 1.9 € kg^−1^ dried product (Alloul *et al.*, 2020). The cost for a packed bag of ROF is taken as 100%, and a relative comparison is made with the MFs. Due to the higher cost of phototrophic organisms, only blends of Spirulina and *Rhodobacter* sp. together with CAB were considered.

### Statistics

The obtained data for the storage tests and plant tests with parsley were statistically analysed with SPSS 24 software and SAS (5 version 9.4, SAS Institute, Cary, USA), respectively. Significance is declared at 95 %, with *P*‐values < 0.05. Normality was checked by Shapiro‐Wilk’s test (applying Bonferroni correction) and homoscedasticity was checked with Levene’s test. When both conditions were met, a one‐way ANOVA with Tukey as *post hoc* test was performed. In case of normal distribution but unequal variances, a Welch’s ANOVA was performed with Dunnett T3 as *post hoc* test. Parameters lacking a normal distribution were subjected to non‐parametric testing according to Kruskal–Wallis with Dunn’s test for pairwise comparisons (applying Bonferroni correction). For analyses that were not performed in triplicate (elemental analysis biomass, petunia fertilization, parsley nitrification rate), no statistical analysis could be performed.

## Conflict of interests

None declared.

## Author contributions

J.S., O.G., E.W, M.S., L.S., H.V., K.F., W.V., G.H. and S.E.V. conceived and designed the experiments. J.S. and O.G. performed the experiments, completed the statistical data processing, interpreted results and prepared figures and tables. G.H. and S.E.V. contributed with the reagents/materials/analysis tools. J.S., O.G. and S.E.V. wrote the manuscript. All co‐authors contributed equally to the revisions of the manuscript.

## Supporting information


**Fig. S1. **Evaluation of general plant health of Petunia (A) at week 4, and (B) at the end of the trial. Scores between 1 and 10. There were no plants with score 10 at the end of the trial. (C) Evaluation of root development 1‐ almost no visible roots 2‐ some visible roots 3‐ well‐developed roots at the bottom of the pot 4‐ good root development all around the pot 5‐ excellent rooting.
**Fig. S2. **pH and EC dynamics in the Parsley growth test in growing media (GM) without fertilizer (control) and with the different fertilizer combinations (ROF = reference organic fertilizer; CAB = consortium of aerobic bacteria; Spirulina = *Arthrospira platensis*, a cyanobacterium; *Rhodobacter* sp. = a purple non‐sulfur bacterium) at a dose of 530 mg N l^−1^ GM.
**Table S1. **Overview of the mixing and packaging cost items used in the cost estimation (source: Greenyard, personal communication).
