# Supplementary material for: Storage, fertilization and cost properties highlight the potential of dried microbial biomass as organic fertilizer
Source: Microb Biotechnol. 2020 Mar 16;13(5):1377–89. doi: 10.1111/1751-7915.13554 (PMC7415357; doi:10.1111/1751-7915.13554)
Supplement: Supplementary file 1 — Fig. S1. Evaluation of general plant health of Petunia (A) at week 4, and (B) at the end of the trial. Scores between 1 and 10. There were no plants with score 10 at the end of the trial. (C) Evaluation of root development 1‐ almost no visible roots 2‐ some visible roots 3‐ well‐developed roots at the bottom of the pot 4‐ good root development all around the pot 5‐ excellent rooting. Fig. S2. pH and EC dynamics in the Parsley growth test in growing media (GM) without fertilizer (control) and with the different fertilizer combinations (ROF = reference organic fertilizer; CAB = consortium of aerobic bacteria; Spirulina = Arthrospira platensis, a cyanobacterium; Rhodobacter sp. = a purple non‐sulfur bacterium) at a dose of 530 mg N l−1 GM. Table S1. Overview of the mixing and packaging cost items used in the cost estimation (source: Greenyard, personal communication). [file MBT2-13-1377-s001.docx]

**Storage, fertilization and cost properties highlight the potential of dried microbial biomass as organic fertilizer**

*Janne Spanoghe^1*^, Oliver Grunert^2*^, Eva Wambacq^3^, Myrsini Sakarika^1^, Gustavo Papini^1^, Abbas Alloul^1^, Marc Spiller^1^, Veerle Derycke^3^, Lutgart Stragier^4^, Harmien Verstraete^4^, Koen Fauconnier^5^, Willy Verstraete^4,6^, Geert Haesaert^3^, Siegfried E. Vlaeminck^1^*

*^*^Equally contributed*

*For correspondence. E-mail siegfried.vlaeminck@uantwerpen.be; Tel. +32 32653689; Fax + 32 32653225.

# Origin and production of the microbial products and the reference organic fertilizer

The CAB biomass was produced in a reactor with an active volume of 150 m³ at the company Avecom (Wondelgem, Belgium). The reactor was a continuously aerated, completely stirred tank reactor, operated at a hydraulic and biomass retention time between 2.5 and 3 days. The biomass was grown on a side stream of a potato processing company (Clarebout, Belgium), i.e. on the process water produced while cutting potatoes. The output of the reactor (containing the nitrogen rich CAB) was first concentrated to a paste of about 10% dry weight by means of a centrifuge or a sieve belt press, and subsequently dried in a vacuum peddle dryer at 70 °C.

The microalga Spirulina was produced by the company AgrAqua (Oosterzele, Belgium) on purified washing water coming from the potato industry, supplemented with struvite and discharge water from an acid air scrubber. The medium was pre-mixed with the other residual water flows and treated in a lava filter depending on the nitritation of the ammonia present in the discharge water. Afterwards the medium was supplemented with an iron chelator and Na_2_CO_3_/NaHCO_3_ as carbon source and pH buffer. Spirulina was grown in raceway ponds in plastic tunnels, dried after harvesting at low temperature (30 °C) and grounded until easily manageable granules were obtained.

For the cultivation of *Rhodobacter* sp., photobioreactors of 2L were used and illuminated with halogen lights. The AT medium was modified and acetic acid, butyric acid and propionic acid were used in a 1:1:1 ratio for a total of 1 g COD.L^-1^ (Imhoff 2006). Subsequently the biomass was oven dried at 70°C.

The chosen ROF (Frayssinet, France), was provided by Greenyard and is made of oil cake and pulp of fruits, composted poultry manure, processed animal proteins containing hydrolyzed feathers, bone and meat powders, horn powder, dried blood and vinasses (conform Regulation (EC) No 1069/2009).

#
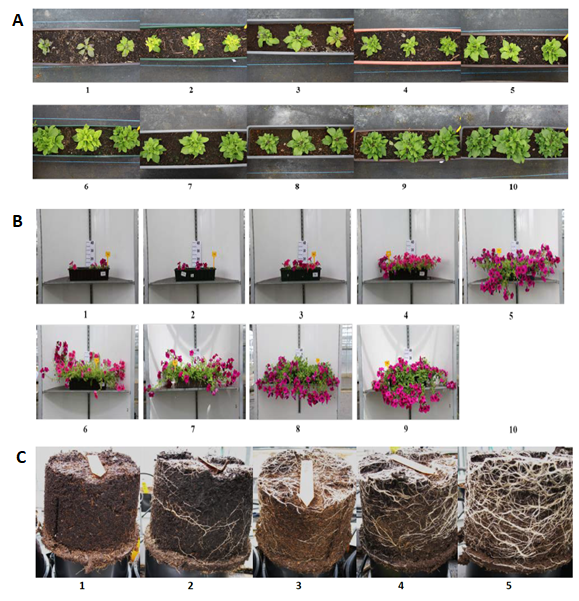


Figure S1: Evaluation of general plant health of Petunia (A) at week 4, and (B) at the end of the trial. Scores between 1 and 10. There were no plants with score 10 at the end of the trial. (C) Evaluation of root development 1- almost no visible roots 2- some visible roots 3- well-developed roots at the bottom of the pot 4- good root development all around the pot 5- excellent rooting


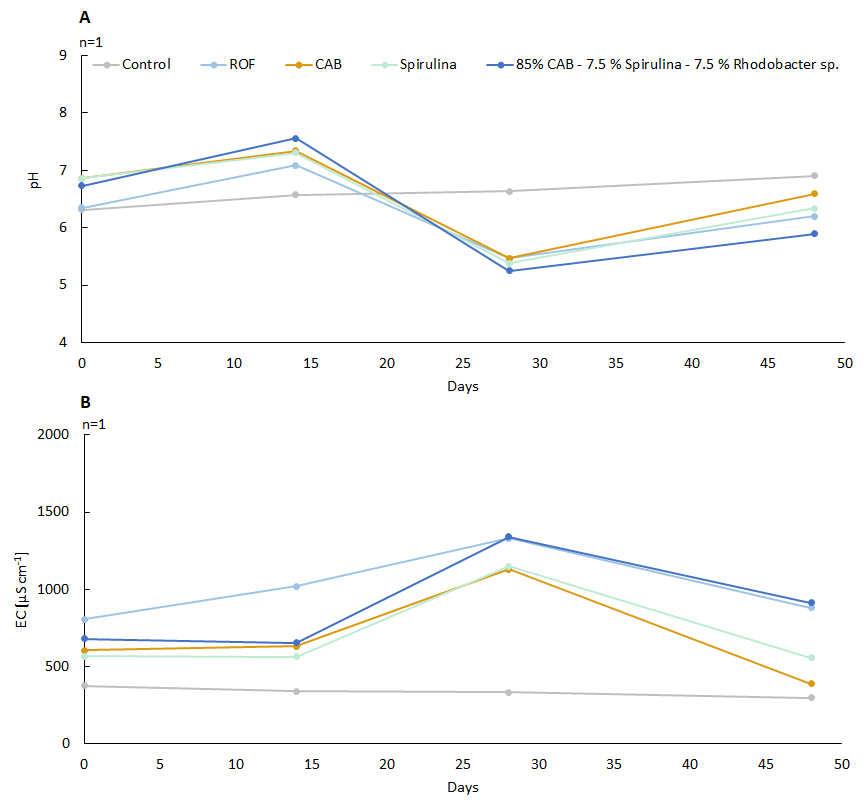


Figure S2: pH and EC dynamics in the Parsley growth test in growing media (GM) without fertilizer (control) and with the different fertilizer combinations (ROF=reference organic fertilizer; CAB = consortium of aerobic bacteria; Spirulina = Arthrospira platensis, a cyanobacterium; Rhodobacter sp. = a purple non-sulfur bacterium) at a dose of 530 mg N L^-1^ GM

Table S1: Overview of the mixing and packaging cost items used in the cost estimation (source: Greenyard, personal communication)

| **Cost item** | **Cost basis** | **Assumptions** | **Cost**  **(EUR/m³ final product)** |
| --- | --- | --- | --- |
| Mixing |  |  |  |
| - Filling ratio | 1.03 m^3^ initial product/m^3^ final product | Packed products are overfilled with 3% to compensate loss of volume |  |
| - Mixing/blending cost | € 4/m³ initial product |  | 4.12 |
| Packaging |  |  |  |
| - Bag | € 0.186/bag | For a bag with 40 L final product | 4.65 |
| - Pallet | € 1.85/pallet | 57 bags per pallet | 0.81 |
| - Hooding | € 1.85/pallet |  | 0.81 |
| - Treatment cost | € 7.6/pallet |  | 3.33 |
| Overhead | 12% | On the mixing and packaging costs | 1.65 |
| Total |  |  | 15.37 |
